# Psychosomatics and mentalization

**DOI:** 10.1192/j.eurpsy.2023.2174

**Published:** 2023-07-19

**Authors:** A. M. Delgado Campos, P. Alcindor Huelva, A. Alvarez Astorga, S. Rubio Corgo, E. Pérez Vicente, M. Arrieta Pey, C. Diaz Gordillo, P. Del Sol Calderón, A. C. Martín Martín

**Affiliations:** Departamento Psiquiatría CSM, Hospital Universitario Infanta Leonor, Madrid, Spain

## Abstract

**Introduction:**

We have investigated the relationship between the Psychosomatic Classification method (Marty) and the Rorschach Test, with respect to the diagnosis of psychosomatic disorders, within the framework of the degree of mentalization measured by both.

**Objectives:**

A) To verify statistical coincidence with respect to the degree of mentalization (risk of generating psychosomatic disorders in a subject) between the Rorschach Test and the diagnostic technique Psychosomatic Classification, by P. Marty. B) To test the hypothesis: Patients diagnosed with infertility, whose degree of mentalization is good, will have a greater probability of achieving a successful pregnancy throught Assisted Reproduction Techniques.

**Methods:**

Two evaluation tools were used: a) Psychosomatic Classification based on the criteria established by this diagnostic method; b) The Rorschach test (based on the evaluation of 29 indicators, selected according to their greater relevance in the generation of somatic symptoms).

A sample of 120 patients (women) diagnosed infertility at the Assisted Reproduction Unit (U.R.A.) at Hospital Universitario 12 de Octubre in Madrid was recruited. The method of ‘statistical correlation of coincidence’ between the results of the two diagnostic instruments used was used. Once both tests had been assessed by the “inter-judge” method and the quantitative values of the selected items had been weighted, the KAPPA statistical method was applied to establish the “correlation of coincidence” between the results of the two assessment instruments.

**Results:**

Considering that the KAPPA method takes values between “0" and ”1" and that between 0.6 and 0.8 the agreement or coincidence is considered good, and above 0.8 very good, the result applied to the hypothesis is 0’76 (’good’).

**Conclusions:**

A) Using the Rorschach Test and P. Marty’s Psychosomatic Classification in a complementary manner, these two instruments together provide high reliability, with respect to the degree of mentalization (a subject’s risk of suffering psychosomatic disorders). B) The degree of mentalization has a significant impact on the success or failure in the application of Assisted Reproduction Techniques in infertile women.

**Disclosure of Interest:**

None Declared

